# Effects of acute aerobic exercise on neural correlates of attention and inhibition in adolescents with bipolar disorder

**DOI:** 10.1038/tp.2016.85

**Published:** 2016-05-17

**Authors:** A W S Metcalfe, B J MacIntosh, A Scavone, X Ou, D Korczak, B I Goldstein

**Affiliations:** 1 Centre for Youth Bipolar Disorder, Sunnybrook Health Sciences Centre, Toronto, ON, Canada; 2 Brain Sciences, Sunnybrook Health Sciences Centre, Toronto, ON, Canada; 3 Heart and Stroke Foundation Canadian Partnership for Stroke Recovery, Sunnybrook Health Sciences Centre, Toronto, ON, Canada; 4 Department of Medical Biophysics, University of Toronto, Toronto, ON, Canada; 5 Department of Physical Sciences, Sunnybrook Health Sciences Centre, Toronto, ON, Canada; 6 Department of Pharmacology, University of Toronto, Toronto, ON, Canada; 7 Hospital for Sick Children, Toronto, ON, Canada; 8 Department of Psychiatry, University of Toronto, Toronto, ON, Canada

## Abstract

Executive dysfunction is common during and between mood episodes in bipolar disorder (BD), causing social and functional impairment. This study investigated the effect of acute exercise on adolescents with BD and healthy control subjects (HC) to test for positive or negative consequences on neural response during an executive task. Fifty adolescents (mean age 16.54±1.47 years, 56% female, 30 with BD) completed an attention and response inhibition task before and after 20 min of recumbent cycling at ~70% of age-predicted maximum heart rate. 3 T functional magnetic resonance imaging data were analyzed in a whole brain voxel-wise analysis and as regions of interest (ROI), examining Go and NoGo response events. In the whole brain analysis of Go trials, exercise had larger effect in BD vs HC throughout ventral prefrontal cortex, amygdala and hippocampus; the profile of these effects was of greater disengagement after exercise. Pre-exercise ROI analysis confirmed this 'deficit in deactivation' for BDs in rostral ACC and found an activation deficit on NoGo errors in accumbens. Pre-exercise accumbens NoGo error activity correlated with depression symptoms and Go activity with mania symptoms; no correlations were present after exercise. Performance was matched to controls and results survived a series of covariate analyses. This study provides evidence that acute aerobic exercise transiently changes neural response during an executive task among adolescents with BD, and that pre-exercise relationships between symptoms and neural response are absent after exercise. Acute aerobic exercise constitutes a biological probe that may provide insights regarding pathophysiology and treatment of BD.

## Introduction

Cognitive dysfunction is common among adolescents with bipolar disorder (BD) during and between mood episodes, contributing to social and functional impairment.^1, 2, 3^ Meta-analytic findings implicate attention and executive function as among the most impaired domains.^2^ Single bouts of aerobic exercise are clinically relevant because they can acutely improve cognition, mood processing, anxiety and smoking cessation,^4, 5, 6^ and because they can inform our understanding of mechanisms underlying the benefits of longer exercise interventions.^7^ Although reviews describe putative benefits of aerobic long-term exercise in BD,^8, 9, 10, 11, 12^ no studies have examined the impact of acute aerobic exercise on neural response during an executive control task in BD.

Numerous studies among adults and youth with BD have examined neural activation for executive processes during sustained attention and inhibition,^13, 14, 15, 16, 17, 18, 19, 20, 21, 22, 23, 24^ using Stop-signal^14, 15, 16, 18, 22^ or Go-NoGo^17, 19, 21, 24^ tasks. Both tasks share a sustained attention component with repeated ‘Go' response to a stimulus and maintenance of vigilance for a Stop cue or a NoGo stimulus. The implementation of these tasks have been varied, namely on the stop trial response instructions, ratios of inhibition trials, block or event analysis, and use of high-level or baseline fixation contrasts.^23, 25^ Consequently, the literature has mixed results. Stop-signal designs are more likely to find latency or accuracy deficits in BDs,^14, 15, 16, 22^ whereas only one study with a Go-NoGo design found any evidence of behavioral deficit.^17^ Brain regions showing difference in neural response between adolescents with BD and healthy controls (HC) were also quite variable. Consistent group differences did emerge in rostral anterior cingulate cortex (rACC),^16, 18, 19, 22, 24^ striatal reward system (caudate, putamen or accumbens)^14, 18, 19, 21, 22, 24^ and ventral prefrontal cortex (vPFC).^14, 16, 21, 22^ However, there is limited consensus in the literature regarding directionality. For the rACC, BDs showed decreased activation^16, 19, 22^ slightly more often than increases.^18, 24^ For the striatum, BDs showed decrease^14, 21, 22^ and increase^18, 19, 24^ equally often. For vPFC, more stability was observed, in the form of decrease.^14, 16, 21, 22^ In the current study, we chose to simplify potential inconsistencies by concentrating on task versus fixation contrasts.^23, 25^

Potential physiological mechanisms of exercise-related reduction of cognitive symptoms in BD include availability of monoamines and endorphins, exercise-induced inflammatory response, reversal of oxidative stress, BDNF, epigenetics, neuroplasticity and cellular resilience.^8, 9, 10, 11, 12, 26^ Studies demonstrating exercise effects on neurocognitive function in BD have been slow to emerge,^11, 12^ however, there is a developing literature establishing improvement of neurocognitive function with long-term exercise in schizophrenia^27, 28^ and depression,^29^ and chronic and acute exercise in ADHD.^30^

Although we know of no studies examining the effects of acute bouts of aerobic exercise on executive function with neuroimaging in psychiatric populations, a recent meta-analysis of healthy participants found moderate intensity exercise improved executive function for individuals of all fitness levels from 14 to 64 years of age when testing occurred at least 11 min after exercise (in contrast to during or immediately after exercise).^31^ One neuroimaging study of acute exercise effects on healthy young female adults demonstrated activation increases in middle prefrontal, lingual and fusiform gyri and decreases in ventral PFC and ACC during an executive task (*n*-back) with stable behavioral performance following acute moderate intensity exercise.^32^ Two studies of acute exercise effects in ADHD using electrical evoked response potential found frontal amplitude changes after exercise for executive tasks.^33, 34^ Two recent studies of chronic exercise effects are also worth mentioning. In the first study, children participating in a 9-month physical activity program demonstrated decreases in dorsal PFC activation but stable ACC activation while improving executive performance (Go-NoGo task).^35^ Finally, one imaging study on chronic exercise effects among adolescents with ADHD found that a combined methylphenidate and exercise program increased frontal lobe activation and decreased temporal lobe activation on a set shifting task after 6 weeks of treatment.^36^

The current study tested the effects of an acute bout of exercise on neural response during sustained attention and inhibition. Based on previous reports, a Go-NoGo design is an optimal choice to minimize performance differences between BDs and HC. We used a high ratio of Go trials to increase sensitivity to the condition most likely to be matched for performance between groups. Two analyses were used. The first, whole brain analysis was used to assess exercise-related change in the two groups while accounting for regional and direction of difference, variability present in previous attention and inhibition studies of BD. The second analysis focused on two core regions that emerged in many of the studies reviewed, the rACC and the striatum; first testing for group differences pre-exercise, and then using these functionally defined loci to examine if exercise diminished or enhanced differences.

We hypothesized baseline and exercise-related differences in rACC, striatal and vPFC regions. Based on the literature review, we predicted that analyzing Go and NoGo scores relative to resting baseline in BD would confirm rACC is a node of deactivation deficit for Go trials; however, the literature did not allow us to anticipate the direction or trial locus of the striatum difference. The existing literature did not clearly suggest an anticipated direction of the exercise-related shift and a goal of this study was to find if acute exercise had positive or negative effect on brain response. Finally, exploratory analyses were used to provide context for symptom effects on rACC and striatal recruitment by testing the relationship of brain activity with mania and depression scores.

## Materials and methods

### Participants

Fifty-four adolescent participants (32 BD and 22 HC) were recruited. BD participants were recruited from the Centre for Youth Bipolar Disorder, a tertiary sub-specialty clinic at Sunnybrook Health Sciences Centre in Toronto. HC participants were recruited from the community via advertisements. Fifty participants (30 BD and 20 HC) were included in the behavioral and imaging analysis after excluding participants for excessive head motion (mean displacement >0.52 mm) or displacement spikes (>2 mm>4% of volumes). The study included English-speaking females and males, ages 13–20 years, who either: (1) met diagnostic criteria for BD I, II or not otherwise specified (symptom severity, comorbidities and current medications of the clinical sample are noted in Table 1); or (2) had no major or recent psychiatric disorders (no lifetime mood or psychotic disorders; no alcohol or drug dependence or anxiety disorders within 3 months) and no family history of BD or psychotic disorder (first- and second-degree relatives). Diagnoses were ascertained during semi-structured interviews with adolescents and parents using the Schedule for Affective Disorders and Schizophrenia for School-Age Children–Present and Lifetime Version (KSADS-PL).^37^ The KSADS Depression Rating Scale (DRS)^38^ and KSADS Mania Rating Scale (MRS)^39^ were used in place of the mood sections of the KSADS-PL. Participants' current illness status was classified as hypomania (mania scores ⩾12), depression (depression scores⩾13), mixed (both scores above threshold) or euthymia (both mania and depression scores below threshold). Full scale IQ as a composite of vocabulary and matrix reasoning scores was tested using the Wechsler Abbreviated Scale of Intelligence (WASI).^40^ Interviewers also administered the Children's Global Assessment Scale (CGAS), which indicates participants' level of general functioning,^41^ and the Family History Screen (with parents and adolescents) to identify the psychiatric status of first- and second-degree relatives.^42^ Participants and their guardians gave written informed consent and all procedures were approved by the institutional Research Ethics Board.

### Procedure

#### Sustained attention to response task

Sustained attention to response task (SART) was used as a Go-NoGo style task.^43, 44^ SART included 180 trials in 30 trial blocks separated by blocks of rest with additional jittered rest between each trial (E-prime v.1.2.1.94, Psychology Software Tools, Pittsburgh, PA, USA). The digits 1–9 were presented in pseudorandom order, with randomized font types and sizes. Each number was displayed for 250 ms followed by 900 ms of fixation. ‘Go' trials consisted of the numbers 1, 2 and 4–9; ‘NoGo' (inhibition) trials consisted of the number 3 (15% of trials). Each run consisted of seven blocks of rest (19.5 s in duration) interleaved with six trial blocks (34.5 s in duration) for a total experimental runtime of 5 min 43.5 s. Data were collected in two sessions, before and 25 min following exercise with pre-experiment practice to reduce learning effects.

#### Exercise

The exercise intervention consisted of 27 min of cycling on a stationary recumbent bicycle-ergometer (5 min of self-paced warm-up, 20 min of aerobic exercise and a 2-min cool down) with the goal of achieving 60–80% of the age-predicted maximal heart rate (HR) during exercise. Age-predicted maximal HR was defined as 220 beats-per-minute minus age in years.^45^ HR was recorded minute-by-minute and participants were instructed to adjust their performance if their HR deviated >5 b.p.m. from the center of their target range (70% of max.).

#### Imaging

Magnetic resonance imaging (MRI) data were collected with a 3 T Philips Achieva system (Philips Medical Systems, Best, The Netherlands) using body coil transmission and an eight channel head receive coil. Structural images were acquired via T1-weighted high resolution fast-field echo imaging (repetition time (TR)/echo time (TE)/inversion time (TI)=9.5/2.3/1400 ms, spatial resolution 0.94 × 1.17 × 1.2 mm, 256 × 164 × 140 matrix, scan duration 8  min and 56 s). Functional gradient echo images with BOLD T2*-weighted contrast were acquired with echo planar imaging (TR/TE=1500/30 ms, flip angle 70°, ascending slices, field of view=230 × 181 mm, spatial resolution 3 × 3 × 4 mm, matrix 76 × 60 × 28, volumes=231).

### Data analysis

#### Demographic and clinical characteristics

Group comparisons were made via independent samples *t*-tests for continuous variables and *χ*^2^-tests for categorical variables.

#### Behavior

Behavioral data was analyzed using SPSS (v.22, IBM, Armonk, NY, USA; *α*=0.05). Minute-by-minute readings of HR were used to calculate participants' total mean HR as a percentage of the estimated maximum for each participant. For interpretability, SART performance measures were analyzed with separate 2 Group (BD, HC) × 2 Session (pre-exercise, post-exercise) univariate analyses of variance (ANOVA). Variables included Go trial % accuracy, correct Go trial reaction time (RT) and NoGo trial error %. To account for correlations among the dependent variables, a two (group) multivariate ANOVA on ▵((pre-exercise)−(post-exercise)) performance was also used to check for differences in pooled variability shared among RT and accuracy.

#### Imaging

fMRI data were analyzed using the FMRIB software library (FSL, v.4.1.9, www.fmrib.ox.ac.uk/fsl). T1-weighted scans were used to co-register individuals to MNI standard space (7 and 12 degrees of freedom) constructed using SyN in ANTs (http://stnava.github.io/ANTs, 12 iterations, buildtemplateparallel.sh).^46, 47^ Functional processing was performed with FSL FEAT, v.5.98 (Analysis Group, FMRIB, Oxford, UK). Pre-processing included motion correction with MCFLIRT, brain extraction with BET, spatial smoothing (Gaussian kernel FWHM=6 mm) and high-pass temporal filtering (60 s). The first 3 volumes were removed to account for T2* equilibration. Volumes with displacement motion spikes>2 mm were de-weighted using unique regressors (<4% volumes in any participant). Intermediate level analysis used to compute ▵((pre-exercise)−(post-exercise)) exercise for individual participants used ordinary least squares models. Group level analyses used mixed effects Flame 1 models. First-level contrasts included correct Go (hereafter ‘Go'), correct NoGo and incorrect NoGo trials separately, minus fixation. Incorrect Go trials were modeled but not analyzed. Activation was assessed for significance at *α*=0.05, with family wise error cluster correction *α*=0.05. Clusters were labeled using Harvard-Oxford cortical and subcortical structural atlases (*P*⩾0.25); stereotaxic coordinates are reported in Talairach MNI space.^48^ Figures are reported in radiological convention (left=right). Whole brain analysis of SART response tested group differences in ▵((pre-exercise)−(post-exercise) exercise effects; analysis of baselines and within-groups differences can be found in Supplementary Materials. Average % signal change from significant clusters for specific Harvard-Oxford labels (*P*⩾0.25) were used to display pre- and post-exercise activation. Regions of interest analyses included bilateral rACC and striatum (caudate, putamen and accumbens) and were defined at the group level using Harvard-Oxford cortical and subcortical structural atlases (*P* ⩾0.25);^48^ rACC was segmented from ACC beginning one section anterior to termination of the hemispheric juncture of the corpus callosum.^49, 50, 51^ Within these ROIs, significant clusters of pre-exercise group differences were identified and peaks were used to display % signal change for pre- and post-exercise.

## Results

### Clinical and aerobic response data

Groups were matched with respect to demographic variables (all *P* >0.08; summary provided in Table 1). Mean HR during exercise was in the middle of the target range (70.0% of age estimated maximum overall; 69.7% BP, 70.5% HC, *P* =0.66). HR exceeded the minimum of the target range for 90% of participants in at least 18 of the 20 min of aerobic activity.

### SART behavioral performance

No univariate group or session differences were observed for Go trial performance or NoGo accuracy. See Table 2 for summary of univariate results and statistical tests. A multivariate ANOVA supported the univariate results (Pillais' Trace =0.085, *F* (3,46)=1.4, *P* =0.249, *η*^2^=0.085, eigenvalue =0.093).

### Whole brain voxel-wise fMRI analysis

Baseline within-group effects, baseline group differences and within-group exercise effects from the whole brain analysis are described in Supplementary Results, Supplementary Tables S1 and S2, and Supplementary Figures S1 and S2. NoGo versus Go and NoGo correct versus NoGo incorrect contrasts were analyzed for baseline group differences and within-group exercise effects for comparison to previous studies with high-level baselines. Significant clusters in these analyses were consistent with those observed in task versus fixation contrasts. The details of these results are described in Supplementary Results.

#### Group effect on ▵exercise brain response

**Figure 1:**
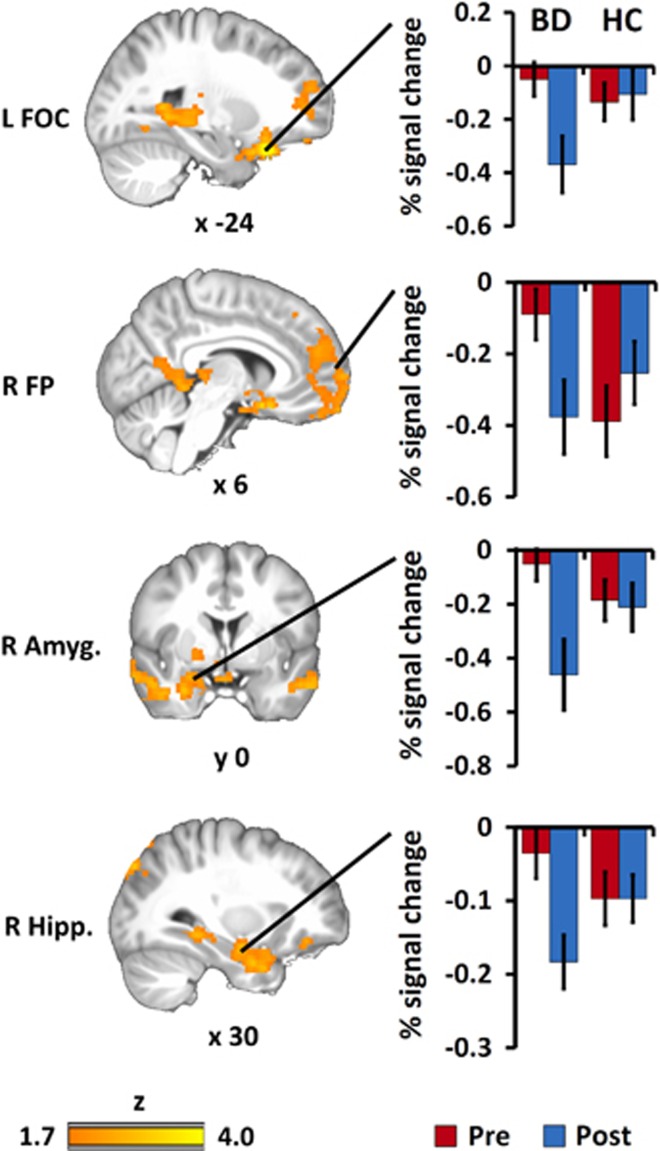
Whole brain group differences in ▵exercise effect for performance-matched Go trial events. Differences in activation in three clusters with peaks in left frontal orbital cortex (FOC; 6623 voxels, max *Z*=4.62, peak=−20, 24, −22), right frontal pole (FP) extending to temporal pole (4028 voxels, max *Z*=3.53, peak=40, 20, −24), and right and left hippocampus (Hipp.) extending to posterior cingulate (2753 voxels, max *Z*=3.55, peak=−16, −42, 2). This deactivation deficit in bipolar disorder (BD) relative to healthy controls (HC) was observed at regional peaks, extending to a relevant subcortical sub-cluster in amygdala (Amyg.). Evidence for deficit was not present following exercise. Error bars ±1 s.e.

For Go trials, a group effect on ▵exercise indicated more change in response to exercise for BDs>HCs including three clusters with peaks at left frontal orbital cortex (FOC; 6623 voxels, max *Z*=4.62, peak=−20, 24, −22), right frontal pole (FP) extending to temporal pole (4028 voxels, max *Z*=3.53, peak=40, 20, −24), and right and left hippocampus extending to posterior cingulate (2753 voxels, max *Z*=3.55, peak=−16, −42, 2). Figure 1 shows profiles of activation averaged across significant voxels separately for left FOC, right FP and right hippocampus, as well as a relevant subcortical sub-cluster in right amygdala. A series of covariate control analyses showed these effects persisted, uncorrected, after accounting for age, ADHD status, exertion level (mean % HR), mania scores, depression scores and BD subgroups (see Supplementary Results for a breakdown of these analyses). No differences were found on NoGo trials.

### Regions of interest fMRI analysis

**Figure 2:**
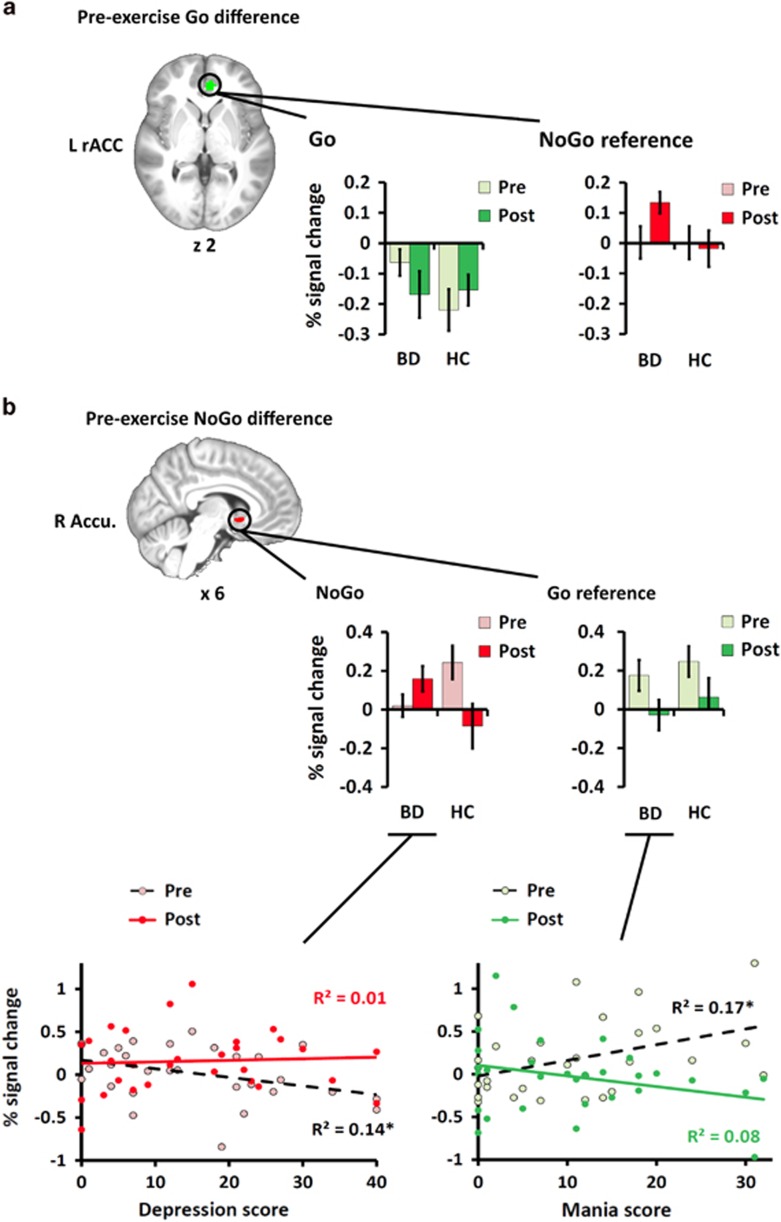
(**a**) Pre-exercise differences found in rostral anterior cingulate (rACC) and (**b**) striatal regions of interest. Left rACC differed by group on Go trials. Bar plots of peak activation before and after exercise showed a pattern consistent with the ventral deactivation deficit in bipolar disorder (BD) relative to controls (HC) found in the whole brain analysis; the pattern was toward reversal after exercise (*P* =0.065). Comparison with NoGo incorrect activation at the same peak showed a different profile with rACC upregulation for NoGo errors after exercise. BDs showed an activation deficit in accumbens (Accu.) relative to HCs for NoGo incorrect trials; the pattern reversed after exercise (*P* =0.005). Similar effects for both groups were observed for Go activation at the same peak. Both sets of activations were compared with BD Mania and Depression scores. There were no effects found for rACC. For accumbens (**b**), higher depression scores correlated with lower NoGo error trial activation prior to exercise but not after; higher mania scores were associated with higher Go trial activation prior to exercise but not after. Error bars ±1 s.e. *P<0.05.

Pre-exercise group differences were found in left rACC (76 voxels, max *Z*=2.45, peak=−8, 44, 0) for Go trials and in right accumbens (15 voxels, max *Z*=2.86, peak=6, 10, −2) for NoGo error trials. Figure 2a shows the pre-exercise difference cluster for Go and the bar plot shows a deactivation deficit for BDs relative to HCs; the pattern was toward reversal after exercise (*F* (1, 48)=3.6, *P* =0.065, *η*^2^ =0.069). For reference, NoGo error signal was also plotted and showed no pre-exercise difference but did increase after exercise. Figure 2b shows the pre-exercise difference cluster for NoGo and the bar plot shows an activation deficit; the pattern reversed after exercise (*F* (1, 48)=8.6, *P* =0.005, *η*^2^ =0.152). Reference to Go signal showed similarly reduced activation after exercise regardless of group. As a covariate control analysis, the effect of medication in relation to Go rACC and NoGo accumbens activation was analyzed. There was a non-significant trend toward greater deactivation deficit in rACC among BD participants taking antipsychotic medications. After accounting for antipsychotic medication, additional medication load was not a factor. Accumbens activity was unrelated to medication status. The details of these analyses are presented in Supplementary Results.

Within the BD group, current mania and depression symptom scores did not correlate with pre-exercise activation in rACC (all *P*>0.05). For accumbens (Figure 2b, bottom), greater depression scores were related to lower pre-exercise activation during NoGo errors (*r*=−0.378, *P*<0.05) and greater mania scores were related to higher pre-exercise activation during Go trials (*r*=0.415, *P*<0.05); neither association was significant after exercise (all *P*>0.05). In addition, illness status (hypomania, depression, mixed symptoms and euthymia) effects at baseline were examined. The subgroup samples were too small to analyze but descriptive data is provided in Supplementary Results. To summarize, BD subgroups showed patterns similar to the primary group, with the exception of weaker trends for depression for rACC and hypomania for accumbens. All groups showed similar exercise effect patterns.

## Discussion

This study investigated the effect of an acute bout of exercise on adolescents with BD to test for positive or negative consequences on neural response during an executive control task. A whole brain voxel-wise analysis of fMRI activation found between-group differences in exercise effects for BDs above HCs during sustained attention throughout vPFC and subcortical structures including the left FOC, and right FP, amygdala and hippocampus. An ROI analysis of baseline activation found pre-exercise group differences for Go trials in left rACC and for NoGo inhibition error trials in right accumbens. Comparison of individual differences in neural activity from rACC and accumbens to mania and depression symptoms for BDs found pre-exercise symptoms were associated with neural activity in accumbens before exercise but not following exercise. Collectively, these results supported our hypothesis that activation differences in response to acute exercise would be larger for BDs relative to HCs.

In the whole brain analysis of Go trials, HCs exhibited deactivation relative to rest before and after exercise. BDs, however, exhibited activity similar to rest, a deactivation deficit relative to HCs that was reversed after exercise. In ROI analysis of baseline Go trial activation, BDs demonstrated a deactivation deficit similar to the broader pattern found in the whole brain analysis. For reference, NoGo activation signal in the same rACC cluster showed no group effects pre-exercise, but did increase in BD after exercise. A different pattern was found for NoGo inhibition errors wherein accumbens showed an activation deficit prior to exercise which was abolished after exercise. For reference, examination of Go activation in the accumbens cluster was similar for both groups, reducing after exercise. Finally, with respect to symptoms, the activation deficit on NoGo error trials was associated with higher depression scores. Mania scores were associated with more activation in response to sustained attention Go trials. After exercise no correlation was found for either measure.

Task performance, demographic variables and exertion were matched between BDs and HCs; in addition a series of covariate control analyses confirmed the stability of ▵exercise effects when accounting for BD subtype and a series of control variables. A secondary goal of the study was to use previously investigated ROIs to better characterize pre-exercise differences in activation both above and below baseline fixation without interpreting subtractive contrasts between attention and inhibition. A third area of interest was to understand how mania and depression symptoms may be related to exercise effects on neural response. Overall the results show that there was a combination of regionally specific deactivation and activation deficits in BD, the latter of which were also related to BD symptoms, with all results indicating reduction of these differences after an acute bout of aerobic exercise.

A previous investigation found that non-medicated adolescents with BD showed elevated ventrolateral prefrontal and superior temporal activity relative to HCs during Go and NoGo trials.^52^ Following 6 weeks of naturalistic pharmacotherapy, the same participants showed elevated hippocampus and thalamus activity during NoGo trials and no cortical differences. In the current study, the majority of our participants were receiving antipsychotic medications (23 of 30) with 27 of 30 receiving at least 1 psychoactive medication of any type. Both rACC and striatal differences between BDs and HCs were observed. Activity in rACC for those not on antipsychotic medications showed trend for more deactivation than those on antipsychotic medication. No medication effects on accumbens were observed. It should also be noted that antipsychotic therapy was related to lifetime severity of symptoms and for both rACC and accumbens, exercise effects for both categories of BD were in the same direction and counter to the direction of change observed in HCs.

Given the elevated risk of vascular pathology associated with BD,^53, 54, 55, 56^ it may be expected that not all acute exercise effects on blood flow-mediated neural response would be beneficial. The consistent pattern instead, is toward normalization of response for BD relative to HC at pre-exercise baseline. Furthermore, a component of the striatal reward system—the accumbens—that was hypoactivated and correlated with BD mood symptoms prior to exercise, was not hypoactivated or correlated with mood symptoms after exercise.

Several explanations could be responsible for the observed effects and changes in availability of monoamines and endorphins, exercise-induced inflammatory response, reversal of oxidative stress, BDNF, epigenetics, neuroplasticity and cellular resilience are all widely discussed pathways for putative post-exercise change in BD symptomology.^8, 9, 10, 11, 12^ Based on regions and timing of response some mechanisms may be more likely than others. For instance, studies with rat models have shown long-term increased exercise is responsible for mood improvement^57^ and increased BDNF in the striatum.^58^ Furthermore, a recent study in BD patients demonstrated that BDNF serum levels increased in females after a single bout of maximal intensity exercise.^26^ Another important pathway could involve monoamines including dopaminergic reward system which terminates in the ventral striatum (that is, accumbens), disruption of which has been implicated as a possible core contributor to BD.^59^ Studies in healthy young adults have demonstrated increases in serum levels of dopamine following acute exercise.^60^ Finally, another class of neurotransmitters which may be even more popularly connected to exercise than dopamine are endorphins, endogenous opiates that enhance mood.^11^ With respect to this investigation, changes in endorphin availability may be especially connected with accumbens function where opiates act as a powerful modulator of dopaminergic reward circuit function in learning and the formation of habit and addiction, as well as mood.^61, 62^

A pervasive finding in this study was that group differences in ▵exercise effects were deactivation deficits in the ventral system found on sustained attention trials. As can be seen in Supplementary Figure 1, dorsal ACC, motor cortex, occipital cortex, parietal cortex and thalamus were all activated during Go trials. Thus it is informative that the only regions sensitive to group effects on ▵exercise were for task-negative active regions. Deactivation profiles during task performance are a hallmark of resting state networks such as the default mode network (DMN), which includes ventral and medial aspects of the PFC and medial and lateral temporal cortices.^63, 64^ The finding of a failure to deactivate core components in this network during sustained attention may be important for interpreting previous findings focusing on the inhibition component of Go-NoGo style tasks in BD.^16, 18, 22, 24^

A recent study of functional connectivity during resting state in BD suggested dysbalance between DMN and other networks may explain core components of depression and mania phases of the disease.^65^ Specifically, in resting state, a deficit in PFC inter-network coupling leads to an over-active DMN and depression symptoms; a deficit in DMN intra-network coupling leads to under-active DMN and mania symptoms. In this context, the deactivation deficit observed here may be interpreted as a signature of this former, DMN dominance effect. Supporting this dysbalance interpretation, adolescents with BD failed to activate the accumbens during NoGo response, a pattern that, following exercise, changed along with the change in rACC deactivation deficit. Accumbens is a key node of the salience network responsible for dynamic switching between DMN and executive functions.^66, 67^ As such, present findings suggest that failure or delay in switching between these functions is potentially a core mechanism in the etiology of the often reported BD inhibition deficit.

When deactivation deficit is used as the basis for a subtractive analysis with inhibition trials this will complicate interpretation of effects^23, 25^ and may place the emphasis of models of BD attention and inhibition dysfunction on the inhibition component of response. The current data suggests ventral and medial PFC regions are implicated in dysfunction of sustained attention associated with a dominant DMN component that persists beyond resting state into an active attention task. Further investigation of functional coupling of DMN and other resting state networks and if the changes in this dysbalance do occur after exercise may be important for better understanding the relationship between mood symptoms and brain response during both attention and inhibition in BD.

Methodological limitations of these findings will now be addressed. There was no session-related control condition, and despite a pre-scanner practice session learning effects remain possible. However, as expected based on the task selected, no significant session effects were observed on behavior. Furthermore, interest in the consequences of acute exercise effects motivated this study, precluding the use of multi-day designs. The current study included a real-life heterogeneous clinical sample, as this is the population in which we foresee aerobic exercise serving as a potential therapeutic approach. Sensitivity analyses that interrogated ADHD comorbidity, current mood symptoms, BD-subtypes and medication status yielded largely convergent findings to the primary analysis (see Supplementary Results). Finally, the influence of pharmacotherapy in this heterogeneous clinical sample must be considered.

## Conclusions

The potential of aerobic exercise as adjunctive therapy in the treatment of adolescent BD is well-known. Here, we endeavored to better understand the effects of an acute bout of exercise on the neurophysiology of a commonly used experimental model of cognitive dysfunction that incorporates executive attention and inhibition processes known to be compromised in BD. We found evidence that a single 20-min bout of aerobic exercise impacts both neural deactivation deficits in attention and activation deficits in inhibition. Improved understanding of the acute effects of aerobic exercise, as well as between-group differences in this regard between BDs and HCs, may inform our understanding of the therapeutic mechanisms of aerobic exercise, as well as unique aspects of neurophysiological response to exercise in BD. Future investigations are needed to better understand the impact of salient clinical characteristics (for example, symptom status) on the observed findings, and, given the brief timeframe of the current study, to determine the duration of the observed effects.

## Figures and Tables

**Table 1:** Demographic and global functioning characteristics among adolescents with and without bipolar disorder (BD) and clinical characteristics of adolescents with BD

	*BD (*n*=30)*	*HC (*n*=20)*	*Statistic*	P*-value*
*Demographics*				
Age	16.8±1.4	16.1±1.5	*t*=−1.8	0.08
Female	17 (57%)	11 (55%)	*χ*^*2*^=0.1	0.91
IQ^a^	109±11.3	113±18.9	*t*=0.97	0.33
Ethnicity (Caucasian)	26 (87%)	16 (80%)	*χ*^*2*^=0.4	0.53
Socioeconomic status	51.1±13.0	55.5±8.1	*t*=1.4	0.18
Lives with both biological parents	16 (53%)	13 (65%)	*χ*^*2*^=0.8	0.41

*Global functioning* b				
Maximum (past year)	63.7±11.8	91.0±3.8	*t*=10.0	<0.001
Current	60.7±10.9	90.8±4.0	*t*=11.8	<0.001

*BD clinical characteristics (*n*= 30)*	Mean±s.d.	n (%)
BD onset age	14.4±2.4	—

*BD subtype:*		
BD-I	—	10 (33.3)
BD-II	—	10 (33.3)
BD-NOS	—	10 (33.3)
Lifetime psychiatric hospitalization	—	13 (54.2%)
		
*Symptom severity*		
Mania^c^	10.9±9.6	—
Depression^d^	14.9±11.9	—
		
*Current illness phase*		
Hypomania^e^	—	5 (16.7)
Depression^f^	—	7 (23.3)
Mixed^g^		8 (26.7)
Euthymia^h^	—	10 (33.3)
		
*Current Medications*		
Second-generation antipsychotic	—	21 (70.0)
Lithium or divalproex sodium	—	6 (20.0)
Psychostimulant	—	2 (6.7)
Antidepressant	—	5 (16.7)
		
*Comorbidity (lifetime)*		
Anxiety disorder	—	19 (63.3)
ADHD	—	14 (46.7)
ODD	—	10 (33.3)
Conduct disorder	—	3 (10.0)
Psychosis	—	9 (30.0)
SUD	—	9 (30.0)

Abbreviations: ADHD, attention deficit hyperactivity disorder; BD, bipolar disorder; CGAS, children's global assessment scale; DRS, KSADS depression rating scale; HC, healthy comparison group; MRS, KSADS mania rating scale; ODD, oppositional defiant disorder; SUD, substance use disorder; WASI, Wechsler abbreviated scale of intelligence.

Variance (±) in s.d. Degrees of freedom for *t*=48 and *χ^2^*=1. For HCs, clinical descriptors were limited to three (15%) individuals diagnosed with ADHD, with two (10%) reporting use of psychostimulants for medication.

a Wechsler abbreviated scale of intelligence.

b Children's global assessment scale.

c KSADS mania rating scale.

d KSADS depression rating scale. ^e^Hypomania, mania scores ⩾12. ^f^Depression, depression scores⩾13. ^g^Mixed, both mania and depression scores above threshold. ^h^Euthymia, both mania and depression scores below threshold.

**Table 2:** Sustained attention to response task performance and ANOVA tables for adolescents with and without bipolar disorder

	*Go RT*	*Go % Acc*	*NoGo % Err*
*Pre-exercise*			
BD	327±79	96±9.5	45±26.1
HC	326±133	95±7.9	44±30.2
			
*Post-exercise*			
BD	319±76	96±6.8	44±23.6
HC	319±127	92±13.1	47±26.5

*ANOVA*	*F (*η^*2*^)	P	*F (*η^*2*^)	P	*F (*η^*2*^)	P
Group	0.1(0.01)	0.991	0.8(0.01)	0.374	0.1(0.01)	0.941
Session	1.0(0.02)	0.334	2.7(0.05)	0.104	0.2(0.01)	0.697
Group × session	0.1(0.01)	0.951	3.6(0.07)	0.065	0.7(0.01)	0.411

Abbreviations: ANOVA, analysis of variance; BD, bipolar disorder; HC, healthy comparison group. Variance (±) in s.d. Degrees of freedom=1, 48.
