# Supplementary figures and images for: Effects of acute aerobic exercise on neural correlates of attention and inhibition in adolescents with bipolar disorder
Source: Transl Psychiatry. 2016 May 17;6(5):e814–. doi: 10.1038/tp.2016.85 (PMC5070058; doi:10.1038/tp.2016.85)

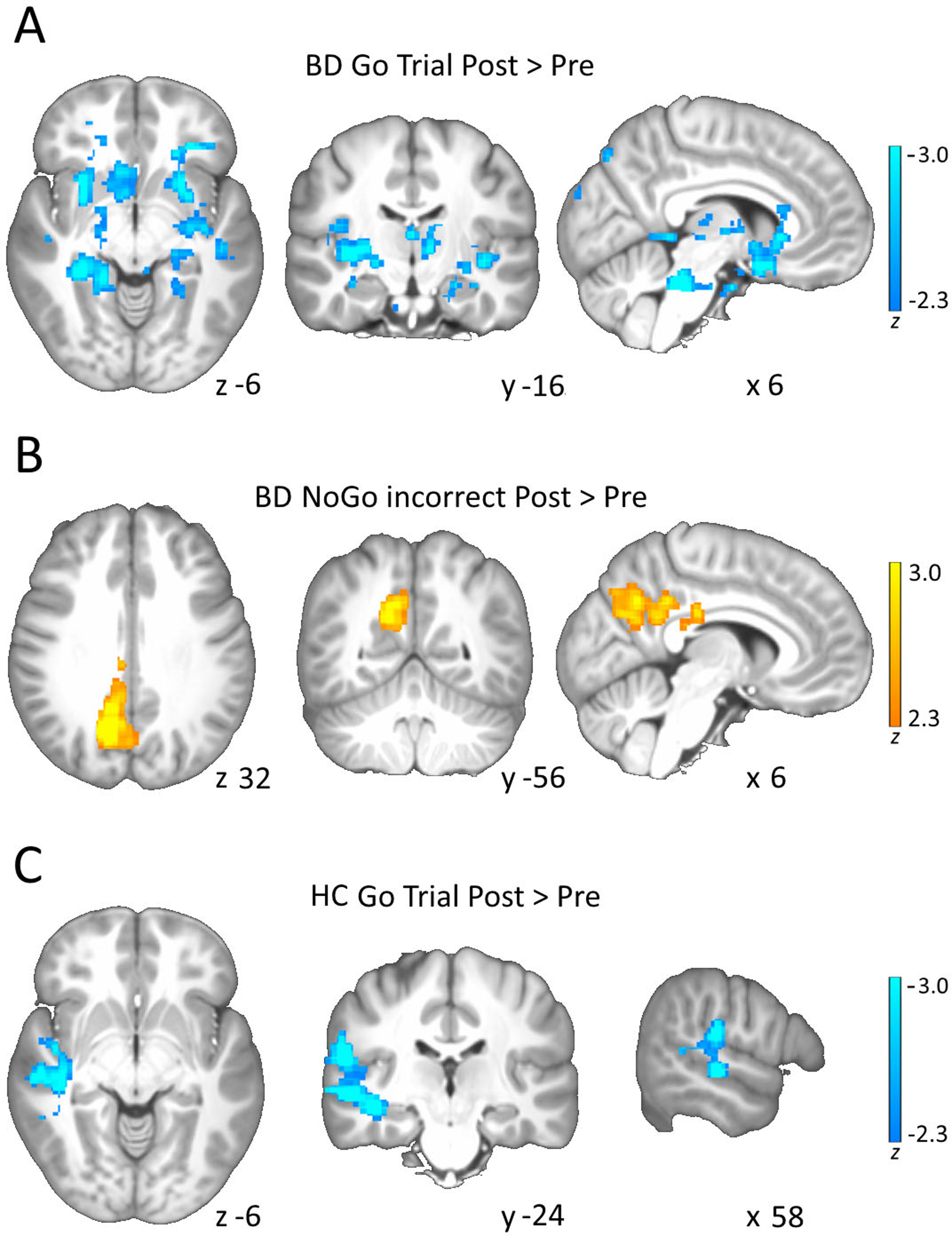

Supplement: Supplementary Figure 2 [file tp201685x2.tif]
