# Supplementary material for: Effects of acute aerobic exercise on neural correlates of attention and inhibition in adolescents with bipolar disorder
Source: Transl Psychiatry. 2016 May 17;6(5):e814–. doi: 10.1038/tp.2016.85 (PMC5070058; doi:10.1038/tp.2016.85)
Supplement: Supplementary Information [file tp201685x3.doc]

**Supplementary**

# Supplementary Methods

## Data Analysis

*Imaging.* Supplementary data analysis was assessed for significance at α=.01 (family wise error (FWE) cluster correction α=.05). Where noted, planned comparisons not significant at α=.01 are reported α=.05, corrected for FWE at *p*<.05 (*k*>629 voxels) based on 10000 Monte Carlo simulations (AFNI-3DClustSim).

# Supplementary Results

## Whole-brain fMRI

### Pre-exercise SART Activation

Within-group effects. Baseline activation was similar for both groups and can be seen in Figure S1. For Go trials (Figure S1-A), the task activated network included bilateral occipital, posterior parietal, sub-cortical, and frontal structures including cingulate, pre-central gyrus, operculum/insula, and left frontal pole. Deactivation compared to rest, included anterior frontal pole, posterior cingulate, and precuneus. NoGo trial (Figure S1-B) activation included dorsolateral prefrontal cortex including middle and inferior frontal gyrus, as well as more rostral aspects of the anterior cingulate; deactivation was present in ventromedial frontal and medial posterior parietal cortex. A contrast of how neural response was different for incorrect NoGo inhibition versus correct inhibition found heightened activation in dorsal ACC leading toward motor cortex and decreased recruitment in accumbens and subcallosal cortex for BDs, but not for HCs (Figure S1-C).

Table S1 presents coordinates and labels for pre-exercise group differences. Go trial differences were found for BD>HC in left peri-insular regions as well as right lateral and medial frontal cortex, HCs showed more activation for NoGo correct and BDs showed more dorsal lateral prefrontal cortex, posterior parietal, and fusiform activation for NoGo incorrect (*α*=.05, FWE corrected). For NoGo correct and incorrect *minus* Go similar patterns were observed as compared to NoGo correct and incorrect *minus* fixation (*α*=.05, FWE corrected). For NoGo incorrect *minus* NoGo correct, HCs showed greater difference in a left dorsal cluster extending from the posterior parietal cortex to the middle frontal gyrus as a result of less activation for incorrect trials (*cf.*Z=40, Figure S1-B Task>Rest: BD=magenta; HC=red) (*α*=.05, FWE corrected).

### Exercise-related change in SART Activation

Within-group exercise effects. Table S2 presents coordinates and labels for exercise effects and Figure 1 presents clusters. For Go trials (Figure S2-A), adolescents with BD showed reduced activation after exercise throughout a ventral network reaching from the occipital cortex to the orbital frontal cortex (OFC); this pattern corresponded with regions of positive activation from pre-exercise within-group effects, especially in striatum. No differences were found for correct NoGo trials. For NoGo incorrect trials (Figure S1-B), precuneus activity increased after exercise, flipping valence from deactivation pre-exercise, to activation after exercise. For NoGo correct and incorrect *minus* Go similar patterns were observed as compared to NoGo correct and incorrect *minus* fixation, with the addition of NoGo incorrect *minus* Go yielding higher activation in the angular gyrus post-exercise. The difference between correct and incorrect NoGo trials decreased after exercise for BDs in precuneus and post-central gyrus (Table S1). In contrast, HC (Figure S1-C) deactivation after exercise was limited to the right ventral parietal and superior temporal cortex. For all other contrasts HCs showed no effect.

A series of control analyses for BDs showed these effects persisted, albeit at lower thresholds, after accounting for age, ADHD status, exertion level as % HR, mania scores, depression scores, and BD subtypes. An F-test of the Session difference amongst BD subtypes for Go incorrect contrasts found differences throughout the clusters showing significant decreases in activation after exercise (*p*<.05, corrected). BD-I had trends for larger, more bilateral medial changes whereas the trend for BD-II and BD-NOS was larger change more laterally. However, follow-up analyses of the Session effects for each separate BD subtype found that the cluster distribution persisted with reduced extent for each subtype (all *p*<.05, uncorrected). An F-test with follow-up tests for NoGo trials revealed a similar pattern as the Go trials, this time with BD-I showing a trend for a larger change, but persistence of the cluster distribution for all subtypes (all *p*<.05, uncorrected). In addition, BD Go and NoGo incorrect Session effects in BD persisted after controlling for individual mania and depression scores (*p*<.05, uncorrected for Go and *p*<.05, corrected for NoGo incorrect). There was no difference after controlling for mean-centered age for the Go or NoGo incorrect contrasts (*p*<.05, corrected). There was no difference after controlling for ADHD status (*p*<.05, corrected). There was no difference after controlling for exertion as mean percent HR (*p*<.05, corrected).

Group effect on ∆ exercise brain response.

There was no difference after controlling for mean-centered age (*p*<.05 uncorrected), nor any difference after controlling for an Age by Group interaction (*p*<.05, uncorrected). There was no difference after controlling for ADHD status (*p*<.05, uncorrected). There was no difference after controlling for mean percent HR (*p*<.05, corrected). The Group by Session effects also persisted after controlling for individual mania and depression scores (*p*<.05, uncorrected). An F-test of the Session difference amongst BD subtypes found differences (*p*<.05 corrected). The trend was for larger vPFC effects for BD-I and -NOS; larger anterior FP effects for -NOS; and larger medial PFC effects for BD-II and -NOS. However, follow-up Group by Session analyses for each separate BD subtype > HCs found that the cluster distribution persisted with reduced extent for each subtype (BD-I *p*<.05 uncorrected; BD-II *p*<.05 uncorrected; BD-NOS *p*<.05 corrected), with exception of partial loss of signal in medial superior gyrus for BD-I.

## Regions of Interest fMRI Analysis

Additional analyses were undertaken to understand pharmacotherapy as it related to activation differences in the rACC and accumbens. First, we divided the sample into those being treated with antipsychotics (AP) (n=23) and those that were not (n=7), and tested for baseline differences in rACC and accumbens signal between groups. BDs on AP medications were more representative of the deactivation deficit pattern in rACC (AP = -0.03; no-AP = -0.16: ns p = .227; HC mean for reference was -0.22). Lifetime symptom mania severity also differed between those receiving (mean=30.3) and not receiving (mean=19.3) APs (*p*=.05). In contrast, accumbens activity deficit during NoGo trials was much more similar for AP (mean = 0.01) and no-AP (mean = 0.05) groups (*p*=.81) (HC mean for reference = 0.24). From the perspective of exercise as a biological probe, exercise effects for both BD subgroups for rACC were toward more deactivation (AP difference = -0.08; no-AP difference = -0.20), in contrast to the pattern observed in HCs toward less deactivation (difference = .07). For accumbens, the BD vs. HC pattern was again observed: AP increase of 0.12 and no-AP increase of 0.21 compared with HC decrease (-0.33). A second analysis examined for correlation between number of medications and baseline rACC and accumbens activation. Twenty-seven of the 30 total BD participants were on at least one psychoactive medication. There was a marginal relationship between medication load and rACC (*r*=.343, *p*=.064), but controlling for the AP effect demonstrated this correlation was heavily weighted toward AP status (*r*=.272, *p*=.154). The accumbens effect was in the opposite direction and not significant (*r*=-274, *p*=.143), and did not change when controlling for AP status (*r*=-.299, *p*=.115).

Potential effects of symptom phases were considered with respect to activity in rACC and accumbens. Although subgroup samples were too small to analyze, we present the data for descriptive purposes. For rACC signal, subgroup means were as follows: hypomania (n=5, mean = -0.06); depression (n=7, mean = -0.18); mixed (n=8, mean = -0.06); euthymia (n=10, mean = 0.01); HCs for reference (mean = -0.22). For accumbens NoGo activation, subgroup means were as follows: hypomania (mean = 0.16); depression (mean = -0.17); mixed (mean = 0.01); euthymia (mean = 0.08); HCs for reference (mean = 0.24). Exercise related change across groups: rACC (hypomania = -0.19, depression = -0.11, mixed = -0.14, euthymia = -0.22); accumbens (hypomania = 0.16, depression = 0.30, mixed = 0.12, euthymia = 0.08,).

## Figure Legends

**Figure S1.** Within-group activation on sustained attention to response task events for those with Bipolar Disorder (BD) and healthy controls (HC). A) Go trial BOLD response. B) NoGo trial BOLD response separated by correct or incorrect. C) Separate contrasts of incorrect NoGo trial BOLD activation and correct NoGo trial activation for BDs and HCs. Incor., incorrect. Co., Correct.

**Figure S2.** Exercise-related change in activation as within-group contrasts for adolescents with Bipolar Disorder (BD) and healthy controls (HC). A) Activation decreased after exercise in ventral aspects of the sustained attention network for BDs (cf. Figure S1-A). B) For BDs, activation increased in the precuneus after exercise and this increase represented pre-exercise deactivation that transitioned to post-exercise activation. Contrasts of the session effect for the difference between incorrect and correct NoGo trials (not pictured) confirmed activation on incorrect inhibition became more similar to correct inhibition activation after exercise for this precuneus area. C) In contrast, HC exercise effect was greater deactivation in an unrelated area after exercise.

| **Table S1.** Neural activation differences at baseline for adolescents with and without Bipolar Disorder. | | | | | | |
| --- | --- | --- | --- | --- | --- | --- |
|  | | Cluster Size (voxels) | Peak  Z-Score | Peak MNI Coordinates (mm) | | |
| Region | Brodmann Area |  |  | X | Y | Z |
|  |  | *Go trials* | | | | |
| BD > HC |  |  |  |  |  |  |
| R Pre-central gyrus/ anterior cingulate cortex | 4/23 | 1476 | 3.7 | 40 | -18 | 52 |
| R Posterior cingulate cortex/ precuneus/ hippocampus | 23/37 | 1430 | 2.9 | 16 | -44 | 24 |
| L Opercular cortex/ middle frontal gyrus | 48/9 | 1204 | 3.7 | -44 | -2 | 18 |
| R Frontal pole | 11/10 | 1075 | 4.6 | 18 | 72 | -4 |
| R Lateral occipital cortex | 39 | 1013 | 3.3 | 50 | -70 | 38 |
| R Inferior temporal gyrus/ middle temporal gyrus | 20/21 | 944 | 3.2 | 68 | -34 | -20 |
| HC > BD |  |  |  |  |  |  |
| No significant clusters | -- | -- | -- | -- | -- | -- |
|  |  | *NoGo correct trials* | | | | |
| BD > HC |  |  |  |  |  |  |
| No significant clusters | -- | -- | -- | -- | -- | -- |
| HC > BD |  |  |  |  |  |  |
| L Inferior frontal gyrus/ orbital frontal cortex | 44/47 | 1455 | 3.7 | -58 | 16 | 16 |
| R Central opercular cortex/ post-central gyrus | 48/3 | 1212 | 3.3 | 22 | -4 | 18 |
| L Middle temporal gyrus | 21 | 802 | 3.1 | -64 | 6 | -26 |
|  |  | *NoGo incorrect trials* | | | | |
| BD > HC |  |  |  |  |  |  |
| L Inferior frontal gyrus/ middle frontal gyrus/ frontal pole | 48/46 | 2694 | 3.4 | -32 | 16 | 24 |
| L Posterior cingulate cortex/ precuneus/ supramarginal gyrus | 23/7/40 | 2536 | 3.4 | -22 | -18 | 46 |
| L Occipital fusiform/ temporal occipital fusiform gyrus | 37/19 | 1638 | 3.0 | -34 | -80 | -16 |
| HC > BD |  |  |  |  |  |  |
| No significant clusters | -- | -- | -- | -- | -- | -- |
|  |  | *NoGo correct minus Go* | | | | |
| BD > HC |  |  |  |  |  |  |
| No significant clusters | -- | -- | -- | -- | -- | -- |
| HC > BD |  |  |  |  |  |  |
| L Frontal orbital cortex/ Inferior frontal gyrus/ Frontal pole | 47/45 | 1133 | 2.9 | -52 | 30 | -10 |
| L Post-central gyrus/ Angular gyrus | 3/39 | 1122 | 3.0 | -56 | -16 | 44 |
| L Middle temporal gyrus | 21 | 912 | 3.3 | -60 | 6 | -26 |
| R Central opercular cortex/ Putamen | 48 | 713 | 3.2 | 22 | -4 | 18 |
|  |  | *NoGo incorrect minus Go* | | | | |
| BD > HC |  |  |  |  |  |  |
| L Inferior frontal gyrus/ middle frontal gyrus/ frontal pole | 48/46 | 1971 | 3.4 | -30 | 20 | 26 |
| L Posterior cingulate cortex/ Precuneus/ supramarginal gyrus | 23/7/40 | 1013 | 3.41 | -32 | -48 | 36 |
| HC > BD |  |  |  |  |  |  |
| No significant clusters | -- | -- | -- | -- | -- | -- |
|  |  | *NoGo incorrect minus NoGo correct* | | | | |
| BD > HC |  |  |  |  |  |  |
| No significant clusters | -- | -- | -- | -- | -- | -- |
| HC > BD |  |  |  |  |  |  |
| L Pre-central gyrus/ precuneus/ middle frontal gyrus/ superior parietal lobule | 3/7/9/40 | 2489 | 3.01 | -22 | -20 | 46 |
| L Frontal pole | 46/10 | 997 | 2.94 | -36 | 52 | 16 |
| R Frontal pole/ superior frontal gyrus | 46/10/9 | 796 | 3.16 | 22 | 36 | 28 |
| L Inferior frontal gyrus/ | 45/47 | 742 | 3.46 | -34 | 20 | 18 |
| BD, adolescents with bipolar disorder. BD, adolescents with bipolar disorder. HC, healthy comparison group. All clusters significant at *p*<.05, cluster corrected for FWE at *p*<.05 (*k*>629 voxels) based on 10000 Monte Carlo simulations (AFNI-3DClustSim). L, left. R, right. B, bilateral. | | | | | | |

| **Table S2.** Differences between pre-exercise and post-exercise neural activation for adolescents with and without Bipolar Disorder. | | | | | | | |
| --- | --- | --- | --- | --- | --- | --- | --- |
|  | | | Cluster Size (voxels) | Peak  Z-Score | Peak MNI Coordinates (mm) | | |
| Region | | Brodmann Area |  |  | X | Y | Z |
| *Adolescents with Bipolar Disorder* |  | |  |  |  |  |  |
|  |  | | *Go trials* | | | | |
| Pre > Post |  | |  |  |  |  |  |
| B Frontal orbital cortex/ putamen/ caudate/ insula | 11/47/48/25/  28/34/35 | | 9884 | 4.5 | 8 | -36 | -28 |
| B Occipital cortex | 19/18 | | 2097 | 4.2 | 28 | -82 | 40 |
| Post > Pre |  | |  |  |  |  |  |
| No significant clusters | -- | | -- | -- | -- | -- | -- |
|  |  | | *NoGo correct trials* | | | | |
| No significant clusters |  | | -- | -- | -- | -- | -- |
|  |  | | *NoGo incorrect trials* | | | | |
| Pre > Post |  | |  |  |  |  |  |
| No significant clusters | -- | | -- | -- | -- | -- | -- |
| Post > Pre |  | |  |  |  |  |  |
| B Precuneus/ R posterior cingulate | 7/23 | | 1484 | 3.8 | 14 | -64 | 32 |
|  |  | | *NoGo correct minus Go* | | | | |
| No significant clusters |  | | -- | -- | -- | -- | -- |
|  |  | | *NoGo incorrect minus Go* | | | | |
| Pre > Post |  | |  |  |  |  |  |
| No significant clusters | -- | | -- | -- | -- | -- | -- |
| Post > Pre |  | |  |  |  |  |  |
| B Precuneus/ posterior cingulate | 7/23 | | 2411 | 3.9 | 16 | -64 | 30 |
| R Angular gyrus | 40 | | 793 | 3.1 | 52 | -50 | 50 |
|  |  | | *NoGo incorrect minus NoGo correct* | | | | |
| Pre > Post |  | |  |  |  |  |  |
| R Precuneus/ posterior cingulate | 7/23 | | 806 | 3.38 | 14 | -66 | 30 |
| R Post-central gyrus | 4/6/3/2 | | 777 | 3.48 | 28 | -28 | 64 |
| Post > Pre |  | |  |  |  |  |  |
| No significant clusters | -- | | -- | -- | -- | -- | -- |
| *Healthy Comparison Group* |  | |  |  |  |  |  |
|  |  | | *Go trials* | | | | |
| Pre > Post |  | |  |  |  |  |  |
| R Supramarginal gyrus/ superior temporal gyrus | 42/22/48/  41/20 | | 1459 | 4.16 | 50 | -38 | 10 |
| Post > Pre |  | |  |  |  |  |  |
| No significant clusters | -- | | -- | -- | -- | -- | -- |
|  |  | | *NoGo correct trials* | | | | |
| No significant clusters | -- | | -- | -- | -- | -- | -- |
|  |  | | *NoGo incorrect trials* | | | | |
| No significant clusters | -- | | -- | -- | -- | -- | -- |
|  |  | | *NoGo correct minus Go* | | | | |
| No significant clusters | -- | | -- | -- | -- | -- | -- |
|  |  | | *NoGo incorrect minus Go* | | | | |
| No significant clusters | -- | | -- | -- | -- | -- | -- |
|  |  | | *NoGo incorrect minus NoGo correct* | | | | |
| No significant clusters | -- | | -- | -- | -- | -- | -- |
| L, left. R, right. B, bilateral. | | | | | | | |
